# Hygienic Kitchens

**Published:** 1907-09

**Authors:** 


**A Monthly Review of Medicine and Surgery.**

*EDITOR:*

WILLIAM WARREN POTTER, M. D.

All communications, whether of a literary or business nature, books for review and exchanges, should be addressed to the editor 238 DELAWARE AVE., BUFFALO, N. Y.

VOL. LXIII. SEPTEMBER, 1907. NO. 2

### Hygienic Kitchens.

DR. ERNEST WENDE, during a former term as health commissioner of Buffalo, rendered the city and indirectly the country at large a great service when he put out of commission the deadly long tube nursing bottle. Almost immediately after the abolishment of this innocent looking but disease breeding tube, infant mortality fell to a nominal rate in this city.

The recent crusade that Health Commissioner Wende has made in the interest of hygienic kitchens is next in importance, in our view, to the long tube nursing bottle campaign. The disclosures made by the health inspectors relating to kitchens and their environment in hotels and restaurants, are such as to create astonishment on the part of the general public, who of necessity patronise them. While it is a comfort to learn that many hotels and other places where food is served were found to be in a cleanly condition yet, on the other hand, to be informed that so many were not only unclean but even filthy, was astounding, some of these too taking rank as first-class establishments.

That serious sickness, more or less wide-spread in character, has not resulted from the neglect disclosed by the inspection, must be more a matter of good luck than otherwise. When it is recalled that in many of these kitchens and ice boxes were found decomposing fish, decaying vegetables, spoiled meats, and tainted milk, one is inclined to exclaim, having in mind the official work of Dr. Wende and his assistants, for this relief, much thanks!

It is scarcely to be comprehended that, in this day and age of improved sanitation and with so many appliances that make for cleanliness and safety, intelligent men and women in charge of kitchens from which food is served to the public should need the stimulus or prodding of sanitary inspectors to compel them to keep their houses in order. Every landlord, steward, or superintendent of a hotel, club, restaurant or other place where food is sold to the hungry traveler, should make daily inspection of his kitchens and other rooms where food is cooked, stored or served, to see that they are kept in perfect sanitary condition and that frequent coats of whitewash are administered to the ceilings and walls.

Scarcely more important than the foregoing is the suppression or elimination of flies, these daily carriers and distributors of infection. We shall not enter into a general discussion of this question at this time, but shall content ourselves with a quotation from an interesting little brochure on Healthy Summer Homes, written by Dr. W. S. Searle, of Brooklyn, in which he deals with this question as follows:

> Still worse as carriers of disease are flies, and they are the most ubiquitous as well as iniquitous pest of this climate, if not in the world. Especially fond of all decaying substances, vegetable or animal, they light and feed upon them, and then, upon our food, carrying all sorts of bacteria and distributing them wherever they go, much as if the farmer should tramp into the parlor in boots that he has worn all day in the barnyard.
>
> Thorough and effective screening, especially of the kitchen and dining room, with death by violence or poison of such as evade our constant vigilance, is absolutely essential to healthy summer homes.

We hope Dr. Wende has given an object lesson to the inhabitants of Buffalo, and especially to the proprietors of hotels, restaurants and eating houses, that will last them a long time. We expect to return to this subject in the near future, giving it more emphasis in detail.

